# Transcriptomic responses of the olive fruit fly *Bactrocera oleae* and its symbiont *Candidatus Erwinia dacicola* to olive feeding

**DOI:** 10.1038/srep42633

**Published:** 2017-02-22

**Authors:** Nena Pavlidi, Anastasia Gioti, Nicky Wybouw, Wannes Dermauw, Michael Ben-Yosef, Boaz Yuval, Edouard Jurkevich, Anastasia Kampouraki, Thomas Van Leeuwen, John Vontas

**Affiliations:** 1 Department of Biology, University of Crete, 71409 Heraklion, Greece; 2 Institute for Biodiversity and Ecosystem Dynamics (IBED), University of Amsterdam (UvA), Science Park 904, 1098 XH Amsterdam, The Netherlands; 3 Department of Crop Protection, Faculty of Bioscience Engineering, Ghent University, B-9000, Ghent, Belgium; 4 Department of Entomology, The Hebrew University of Jerusalem, Rehovot 76100, Israel; 5 Department of Plant Pathology and Microbiology, The Hebrew University of Jerusalem, Rehovot 76100, Israel; 6 Institute of Molecular Biology & Biotechnology, Foundation for Research & Technology Hellas, 100 N. Plastira Street, GR-700 13, Heraklion Crete, Greece; 7 Laboratory of Pesticide Science, Department of Crop Science, Agricultural University of Athens, 11855-Athens, Greece

## Abstract

The olive fruit fly, *Bactrocera oleae*, is the most destructive pest of olive orchards worldwide. The monophagous larva has the unique capability of feeding on olive mesocarp, coping with high levels of phenolic compounds and utilizing non-hydrolyzed proteins present, particularly in the unripe, green olives. On the molecular level, the interaction between *B. oleae* and olives has not been investigated as yet. Nevertheless, it has been associated with the gut obligate symbiotic bacterium *Candidatus Erwinia dacicola*. Here, we used a *B.oleae* microarray to analyze the gene expression of larvae during their development in artificial diet, unripe (green) and ripe (black) olives. The expression profiles of *Ca. E. dacicola* were analyzed in parallel, using the Illumina platform. Several genes were found overexpressed in the olive fly larvae when feeding in green olives. Among these, a number of genes encoding detoxification and digestive enzymes, indicating a potential association with the ability of *B. oleae* to cope with green olives. In addition, a number of biological processes seem to be activated in *Ca. E. dacicola* during the development of larvae in olives, with the most notable being the activation of amino-acid metabolism.

The olive tree, *Olea europaea*, is one of the most important fruit trees cultivated in the Mediterranean basin since ancient times and more recently, in additional regions in the world^1^. Olive tree orchards cover more than 10 million hectares, and the olive production in 2013 exceeded 20 million tons per year^2^. The olive fruit fly *Bactrocera oleae* (Diptera:Tephritidae) is the most destructive olive pest. Infestation by *B. oleae* has been recorded in regions around the Mediterranean Sea, but also in Central and South Africa, Near and Middle East, and Central America and California^3^. The female fly lays its eggs in the olive fruit, and the newly hatched larvae feed upon the fruit mesocarp. During feeding, the larva forms tunnels inside the drupe destroying the pulp and enabling secondary infestation by bacteria and fungi. This severely reduces the quality of the olive oil, as well as the production of table olives, since these become unsuitable for consumption^3^.

*B. oleae* larvae are strictly monophagous and are one of the few insect larvae capable of feeding on olive mesocarp. In contrast to other frugivorous Tephritidae Diptera, such as the medfly *Ceratitis capitata* and the oriental fruit fly *Bactrocera dorsalis* (Hendel), which feed upon hydrolyzed compounds of decaying and ripe fruit, *B. oleae* has the unique ability to utilize olive proteins and other nutrients of the olive flesh, as well as cope with high levels of phenolic compounds, which can reach up to 14% of the dry fruit weight, particularly in the unripe (green) olives^4^,^5^,^6^. The phenolic composition of the olive is complex, and varies depending on the variety, maturation stage, season, geographical region, and cultivation practices^6^. Oleuropein is the most abundant phenolic compound in the olive fruit, representing the major constituent of unripe, green olives^7^,^8^. During maturation, as the olive fruit changes color from green to black, the phenolic compound content is also substantially reduced (reviewed in Conde *et al*.^9^). The underlying mechanisms that allow *B. oleae* to overcome the high content of phenolic compounds and utilize the olive flesh have not been fully elucidated as yet. The genome of *B. oleae* is not sequenced and thus, any special features that might have evolved and associate with this ability have not been investigated. To date, the transcriptome of *B. oleae* has been sequenced by 454 pyrosequencing^10^, and transcriptomics have been employed for the investigation of different aspects of *B. oleae* biology such as spinosad resistance^11^, the differences between female and male reproductive systems and differential expression of olfactory genes^12^. However, there are no studies investigating the transcriptional response of the olive fruit fly larvae upon feeding on olives.

One explored aspect of the interaction, known from earlier pre-omic studies, is that the ability of the *B. oleae* larvae to develop on olives is associated with the presence of gut symbiotic bacteria, which appear absolutely necessary for the larval development in green olives, but not in black^13^,^14^,^15^,^16^. It has been proposed that the symbiotic bacteria may provide the olive fruit fly with some advantage related to survival within the harsh environment of unripe olives, either by detoxifying phenolic compounds (e.g. oleuropein) or by being involved in the enzymatic hydrolysis of dietary proteins and the synthesis of amino acids directly related to nutrient availability, which is limited in green olives^13^,^17^,^18^. Evidence for this hypothesis comes from two recent studies, indicating a role for the gut bacterial community in overcoming host defense and in nitrogen source utilization^13^,^19^. It is believed that the above advantages are attributable to one bacterial species, the taxonomic identity of which has been a controversial issue: For example, Kounatidis *et al*. had identified the symbiont to be *Acetobacter sp.*^19^, but the current consensus points towards the unculturable species *Candidatus Erwinia* dacicola^20^, first observed by Petri in 1909^21^ and initially assigned to the species *Pseudomonas savastanoi. Ca. E. dacicola* is an obligate symbiont, maternally transmitted, and tightly associated with *B. oleae* through all its life stages. It represents the largest fraction of the gut microflora of *B. oleae* and is primarily, but not exclusively, detected in the digestive system of larvae^22^. *Ca. E. dacicola* is common and widespread within olive fly populations from several geographical regions^13^,^19^,^22^,^23^,^24^.

The interaction between *B. oleae* and olives has been studied from the plant perspective, whereby transcriptomic and proteomic approaches were used for the analysis of the molecular response of olive fruits attacked by *B. oleae*^17^,^25^. These studies showed upregulation of genes involved in the production of reactive oxygen species (ROS), activation of stress response pathways, as well as production of compounds involved in direct anti-herbivore plant defense. This latter group includes trypsin inhibitors (trypsin protease inhibitor II, trypsin/chymotrypsin inhibitor), which may inhibit normal herbivore assimilation of plant protein. In contrast, the molecular mechanisms that the *B. oleae* larvae and/or its major bacterial symbiont *Ca. E. dacicola* have evolved as an adaptation to the olive flesh have not been studied yet, due to the lack of genomic information and molecular tools for both species.

In this study, we constructed an Agilent microarray chip based on the previously published *B. oleae* transcriptome^10^ and analyzed the gene expression of wild larvae during their development in artificial diet and in green and black olives in the presence of symbiotic bacteria. In parallel, we sequenced, using MiSeq technology, gut samples from *B. oleae* larvae feeding on olives and used the data to obtain the first transcriptome assembly of the symbiont and compare the expression profiles of *Ca. E. dacicola* genes during larval development in green and black olives.

## Results and Discussion

### *Bactrocera oleae* contigs differentially expressed between larvae developing in olives and artificial diet

**Figure 1:**
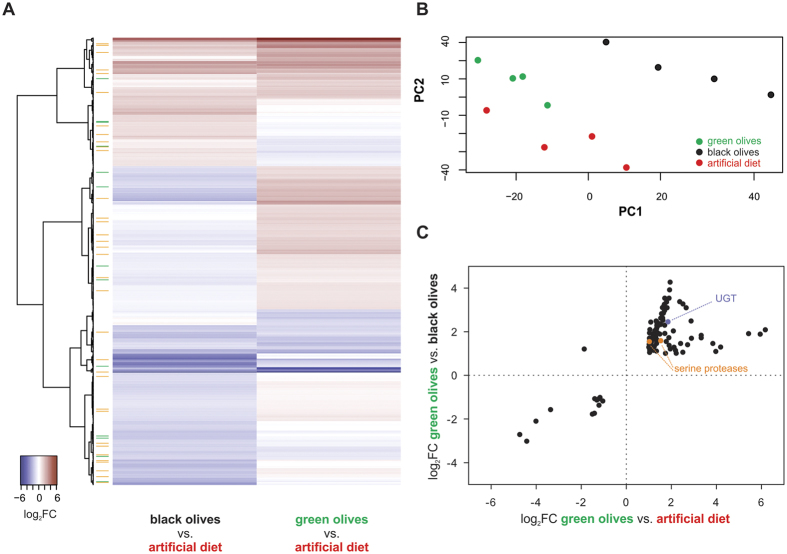
(**A**) Heatmap plot showing gene expression level ratios (relative to artificial diet) for *B. oleae* differentially expressed genes (n = 616) in the two olive regimes of this study. Orange and green sidebars indicate genes that code for enzymes predicted to digest food and detoxify plant metabolites, respectively. Clustering of the genes (Euclidean distance, Ward) was based on their relative transcript levels in the two transcriptomic comparisons. (**B**) PCA plot showing the four biological replicates of each feeding regime: black and green olives and artificial diet. PC1 and PC2 represent 30.00% and 20.04% of the total data variance, respectively. The PCA analysis was performed on the transcript levels of 17,797 genes *B. oleae* showing an FDR-corrected p¬-value < 0.05 in at least one feeding condition. (**C**) Scatterplot of significantly differentially expressed genes (n = 108) in both larvae feeding on green olives *vs*. artificial diet and green olives *vs*. black olives transcriptomic comparisons. Contig04001 is predicted to code for a UDP-glycosyltransferase (UGT) (indicated in blue). The contigs 04849 and 01887 are predicted to code for digestive serine proteases (indicated in orange).

**Figure 2:**
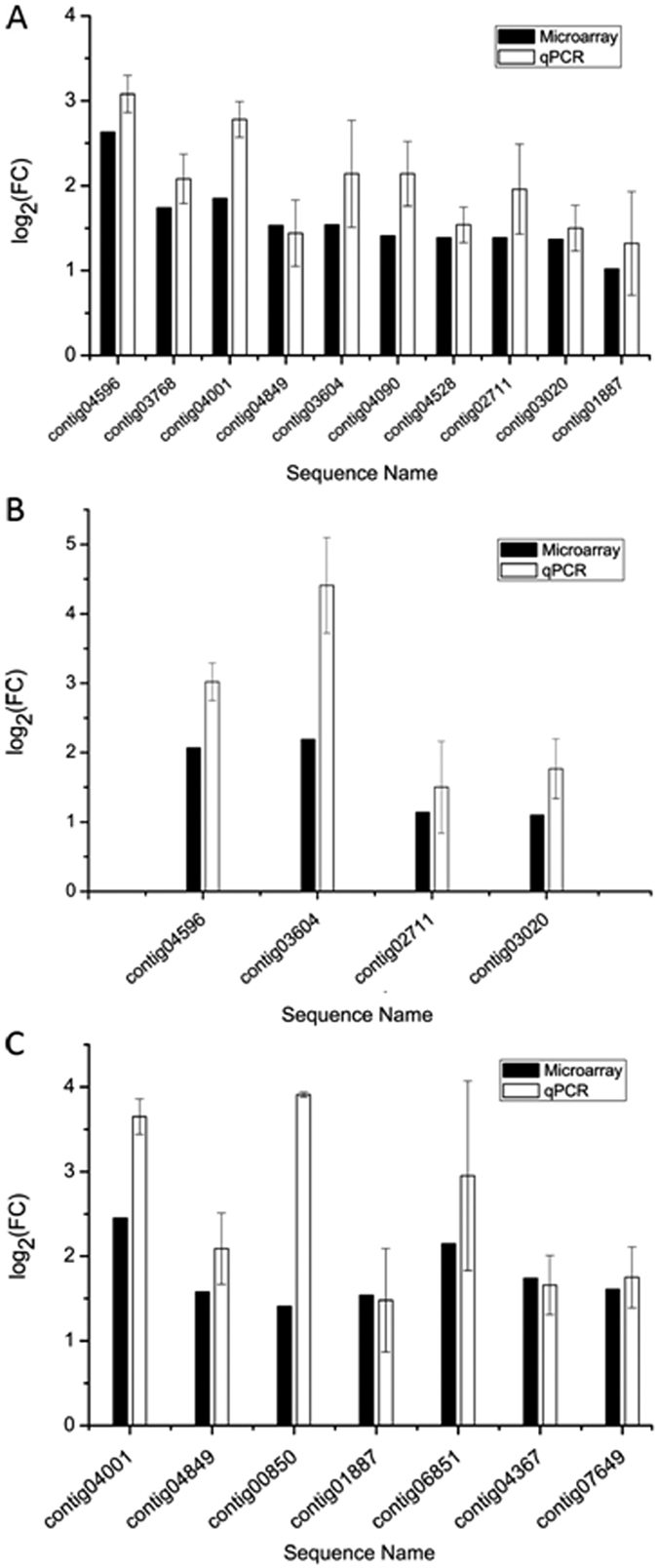
Microarray validation of selected putative detoxification and digestive genes overexpressed in the 3 transcriptomic comparisons. (**A**) Larvae fed upon green olives *vs*. larvae fed upon artificial diet. (**B**) Larvae fed upon black olives *vs.* larvae fed upon artificial diet. (**C**) Larvae fed upon green olives *vs.* larvae fed upon black olives. Error bars represent the standard deviation of four biological replicates. Microarray data are also presented. Contig04596: serine protease; contig03768: serine protease; contig04001: UDP-glucosyltransferase; contig04849: serine protease; contig03604: cytochrome P450; contig04090: serine protease; contig04528: serine protease; contig02711: serine protease; contig03020: N-acetyltransferase; contig01887: peptidase; contig00850: metalloprotease; contig06851: lysozyme; contig04367: serine protease; contig07649: protease.

In order to identify genes that are associated with the unique ability of *B. oleae* among tephritid flies to develop in olives, three comparisons were performed in four replicates, each using the Agilent microarray platform (Fig. 1). Differences in transcript expression in larvae fed on green olives, black olives, and artificial diet, were recorded in all possible comparisons. Differential expression of selected genes was confirmed by RT-qPCR (Fig. 2). A two-dimensional PCA plot of the transcriptomic signals separated the three groups of larvae based on their feeding regime (Fig. 1B). Replicates within the green olive treatment clustered more closely together, while the other two treatments showed a higher intragroup variability. Using artificial diet as the reference, larvae showed a partially shared but also unique transcriptomic response to green and black olive feeding (Fig. 1A). Arthropod herbivores commonly exhibit a differential transcriptomic profile in response to different diets. These metabolic changes typically include detoxification and digestive pathways and are thought to allow the herbivore to survive the unique dietary compositions^26^,^27^. Indeed, many differentially expressed genes are predicted to code for detoxifying and digestive enzymes (Fig. 1A, green and orange labels, respectively). The profiles observed in this study may reflect different feeding challenges associated with the two olive maturation stages. Green olives are considered more toxic because of the higher levels of phenolic compounds they contain (e.g. oleuropein), while in black olives, the phenolic levels are reduced^4^,^5^,^6^ and proteins are hydrolyzed and, in general, more available. In unripe, green olives oleuropein cross-links proteins while destroying their lysine residues, thus reducing protein and lysine availability for the larvae^28^,^29^.

**Figure 3:**
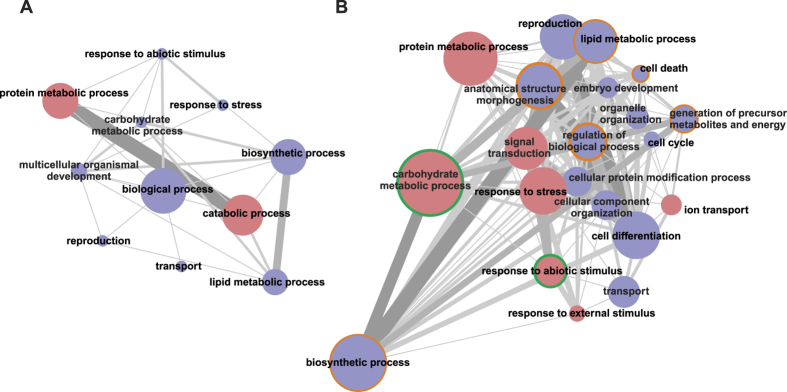
Gene set analysis of the biological processes coded by the differentially expressed genes in *B. oleae* larvae feeding on green olives, (**A**) compared to larvae reared on artificial diet and (**B**) black olives (log_2_FC > 1 and adjusted *p-value* < 0.05). Gene sets with gene members between 5 and 30 were used for analysis. Nodes and interconnecting lines represent gene sets and the inter-set overlap, respectively. Using larvae fed on artificial diet or black olives as the reference, blue and red node colors indicate that the gene members were under and over-expressed in the larvae feeding on green olives, respectively. The significantly enriched gene sets are indicated by an orange halo (BH-adjusted *p-value* < 0.05, obtained through piano) analysis and a green halo (Fisher’s exact test, BH-adjusted *p-value* < 0.05, obtained through Blast2GO analysis). The number of genes on the interconnecting lines ranged from 1 to 9 and 1 to 13 in panel A and panel B, respectively. Gene sets are named by their descriptive labels. The gene set map and directionality of differential expression was produced using the piano package^66^. For more a detailed list of GO-enriched terms, see Table S4.

Specifically, we identified 215 contigs that were differentially transcribed between larvae fed upon green olives and artificial diet (Fig. 1A, Table S3). An overview of all GO terms associated with these contigs is shown in Fig. 3A and in Table S4. Out of the 215 differentially transcribed contigs, 77 coded for proteins with a hit in NCBI database. One hundred and fifty seven contigs (of which 41 with a blastx description) were over-expressed and 58 (of which 36 with a blastx description) were under-expressed. Among the 41 over-expressed contigs with a blastx description, several contigs encoding for enzymes related to food digestion and detoxification of xenobiotics were identified. Nine putative serine-type proteases, such as midgut trypsins (e.g. contig04090, contig05378), as well as other proteases and a maltase (contig02414) were over-expressed in larvae developing in green olives. The induction of peptidase activity was also confirmed by GO enrichment analysis (Table S4). The increased production of digestive enzymes may allow digesting proteins of the olive flesh, but may also serve as a defense mechanism to counteract the negative effects of trypsin inhibitors induced by the olive fruit upon *B. oleae* attack^25^. Four putative detoxification genes, encoding for a UDP-glucosyltransferase (contig04001), a cytochrome P450 monooxygenase (contig03604), a N-acetyltransferase (contig03020) and a glycine methyltransferase (contig03118) were over-expressed upon development in green olives compared to artificial diet; these detoxification genes could be involved in the de-activation of phenolic compounds present in the green olive flesh.

In the next transcriptomic comparison, a total of 185 contigs (of which 86 with a blastx description) were found as differentially expressed between larvae developing in black olives compared to larvae developing in artificial diet (Fig. 1A, Table S5). Sixty-three contigs (of which 22 with a blastx description) were over-expressed, while 122 (64 with a blastx description) were under-expressed. Among the over-expressed contigs coding for proteins with a predicted function, 2 serine proteases were identified (contig04596, contig02711) and 5 contigs related to detoxification: 2 contigs encoding for a P450 monooxygenase (contig03604, contig10157), 2 encoding for a GST (contig09042, contig07812), and one encoding for a N-acetyltransferase (contig03020). Over-expression of some of these genes was also confirmed by RT-qPCR (Fig. 2B). The contigs 04596 an 02711 (encoding for putative serine proteases), 03604 (encoding for a putative P450) and 03020 (encoding for putative N-acetyltransferase) were also found over-expressed in the microarray comparison of larvae fed upon green olives *versus* artificial diet, indicating a possible association of these contigs with the adaptation of the olive fruit fly to olives. Using the artificial diet as the reference feeding condition, the number of significantly over-expressed contigs upon development of larvae in black olives (n = 63) was lower than that of larvae developing in green olives (n = 157). This indicates that the number of processes activated in the insect during growth in the black olives may be closer to the number of processes activated during artificial diet growth, possibly because the two diets are quite similar (for example, both containing hydrolyzed proteins), while the green olive environment seems to induce a number of additional genes/processes, possibly due to its demanding nature (for example, requiring detoxification and absorption of limited-availability nutrients).

### Differentially expressed contigs between *Bactrocera oleae* larvae feeding on green immature olives compared to larvae feeding on mature black olives

In order to identify genes that are associated with the ability of *B. oleae* to specifically develop in the green olive, gene expression levels of larvae fed upon green olives were compared to those of larvae fed upon black olives. A total of 456 contigs were differentially expressed in larvae fed upon green olives compared to larvae fed upon black olives (Table S6). Gene set analysis of the biological processes associated with these genes is presented in Fig. 3B. From the 456 contigs, 245 encoded genes with a known function based on similarity searches using blastx. Three hundred and fifty six contigs (of which 183 with a blastx description) were over-expressed in green olives, while 100 contigs (of which 62 with a blastx description) were under-expressed. The expression of selected genes found as differentially transcribed between *B. oleae* larvae feeding on green immature and black mature olives was confirmed by RT-qPCR (Fig. 2C).

A large set of genes (n = 24) predicted to code for enzymes involved in digestion were over-expressed. Specifically, contigs encoding for putative lysozymes (contig06851, contig08507), numerous proteases (including serine proteases, e.g. contig04367, contig04849, contig06115, contig04313, contig01887) and metalloproteases (e.g. contig04956, contig00850), a phospholipase (contig04753), nucleases (e.g. 00776, contig01101, contig01241) and the digestion facilitator peritrophin (contig06046) were over-expressed in larvae fed on green olives, compared to those fed on black olives. The over-expression of a selection of these genes was confirmed by RT-qPCR (Fig. 2C). The over-expressed genes related to digestion represent 8.6% of the over-expressed genes with a predicted function, suggesting that the activation of the related pathways for the digestion of dietary proteins may be an important mechanism employed by *B. oleae* larvae during development in green olives. Except from protein digestion, the induction of peptidase activity may relate to the adaptation of the olive fruit fly to olives *via* protein degradation as a means to meet energy demands during stress^30^ or protein biosynthesis and modification of enzymatic conformation in the context of processes induced by *B. oleae* to respond to plant defense^31^. Moreover, the elevated peptidase activity may represent a mechanism to counteract the negative effects of protease inhibitors produced by olives upon larval attack^25^.

Interestingly, 6 genes predicted to encode for enzymes commonly associated with detoxification of xenobiotics were also over-expressed, namely a putative ABC transporter (contig03867), an alpha-esterase (contig02152), 3 UDP glycosyltransferases (contig04001, contig01274 and contig05006) and a beta-galactosyltransferase (a type of glycolytransferase, contig06187). The detoxification enzymes encoded by these genes may play a role in the ligation or sequestration of phenolic compounds, abundantly present in green olives. The over-expression of a set of glycosyltransferases is in line with other studies that associate the detoxification of plant phenols by glycosylation. For example, BmUGT1 was associated with elimination of the effects of ingested plant phenols in *Bombyx mori*^32^, beta-glycosylation by beta-glycosyltransferases was correlated with detoxification of toxic phenols present in *Manduca sexta* larval diet^33^, while recently, the detoxification of gossypol was associated with UGT41B3 and UGT40D1 in *Helicoverpa armigera* and *Heliothis virescens*^34^.

Contigs with blastx description related to lipid metabolic processes were found to be significantly under-expressed in green olive fed larvae compared to black olive- fed ones (Fig. 3B and Table S5). Increased lipid metabolism in black olives possibly reflects the high oil content of black olives; it is well known that during olive maturation oil synthesis increases (reviewed in Conde *et al*.^9^).

Using the different transcriptomic profiles, we were able to identify those genes that uniquely and consistently responded to green olive exposure (Fig. 1C). Among them, two putative serine proteases (contigs 04849 and 01887) and one predicted UGT (contig04001) were found to be consistently overexpressed in larvae fed upon green olives (Fig. 1C). This may indicate their crucial role in digestion and detoxification of green olive compounds.

In conclusion, several genes were found overexpressed in the olive fly larvae when feeding in green olives. Among these, a number of genes encoding detoxification and digestive enzymes, indicating a potential association with the ability of *B. oleae* to cope with green olives, in line with other insect mechanisms that have been developed to overcome two-component chemical defense in plants, and specifically b-glucosidase activated phytochemicals (such as oleuropein)^35^.

### The draft transcriptome of *Ca. E. dacicola* reconstructed from larval gut samples

We next compared two stages of olive fruit ripening, green and black, from the bacterial point of view, by studying gene expression patterns of the olive fruit fly symbiont *Ca. E. dacicola*. For this purpose, we first reconstructed the transcriptome of *Ca. E. dacicola* from a combined pool of RNA-seq reads coming from the two stages. In order to maximize the number of sequences of symbiont origin, we isolated RNAs from gastric caecas, specialized appendages of the insect’s midgut, in which the symbiont cells can be found in high concentrations at the larval stage^22^. RNAs from gastric caecas of 3^rd^-instar olive fruit larvae developing in green and black olives were used to construct two stranded, ribosomal-depleted and random-primed libraries, sequenced with MiSeq Illumina technology. Because these reads represent a mixture of sequences from diverse bacteria inhabiting the gastric caeca and sequences from insect tissue, we identified, by means of taxonomical mapping (see Methods), a fraction of reads that could be safely assigned to the genus *Erwinia*. This was the most dominant fraction among the taxonomically mapped microbial reads; we also checked for the presence of *Acetobacter sp*. in our sequenced samples, and these were negligible to null (full taxonomic classification results presented in Table S7). The reads identified as ‘*Erwinia*’ from both conditions represented 14.6% of the initial reads, and a total of 510 Mbp. Using this fraction, we *de novo* assembled the *Ca. E. dacicola* transcriptome with the program rnaSPAdes^36^,^37^ and performed an automatic annotation using RAST^38^. To further confirm that our approach of selecting *Erwinia* reads from taxonomic mapping is successful, we aligned the longest 16S predicted sequence from our annotated transcriptome assembly (deposited in GenBank with accession number: KX388537) to other published 16S rRNA sequences of *Ca. E. dacicola*. The nucleotide alignment showed that our sequence is 99% similar to 16S sequences of *Ca. E. dacicola* from Italian and Spanish strains^20^,^39^ (Moret *et al*., unpublished data with accession numbers FM958430.1 and FM958428.1) (Figure S1). Note that since our partial 16S sequence comes from assembly of *Ca. E. dacicola* reads obtained from two distinct larval feeding environments, it cannot be excluded that the differences it presents to the published sequences may relate to its multi-isolate nature (different isolates may inhabit the gastric caeca of green- and black olive-fed flies).

Overall, our approach of pooling bacterial RNAs derived from different stages of olive fruit ripening allowed to recover a considerable fraction of genes of *Ca. E. dacicola*: The assembled transcriptome of the symbiont (Table 1, Table S8) covers approximately 2.7 Mbp and contains 3,206 predicted protein-coding genes (ribosomal RNA genes are not presented here, because we consider predictions on their number unreliable in the context of sequencing rRNA-depleted libraries). The number of *Ca. E. dacicola* protein-coding genes is lower than the number of coding genes predicted by the same platform, RAST, for sequenced genomes of other *Erwinia* species, such as *E. toletana*^40^ (3,709 genes) and *E. oleae*^41^ (4,619 genes). Similarly, the number of tRNAs encoded is smaller (44, with genomes of other *Erwinia* species commonly encoding between 65 for *E. tracheiphila*^42^ and 81 for *E. tasmaniensis*^43^). However, the endosymbiont is expected to have a reduced gene set compared to free-living species of the same genus, as observed for other maternally-transmitted insect symbionts (reviewed in McCutcheon and Moran^44^). We cannot exclude that the transcriptome assembly of *Ca. Erwinia dacicola* is to some extent incomplete; additional sequencing in the future will allow assessing the completeness of our assembly. RAST gene predictions appeared rather fragmented (Table S8), with a number of short genomic elements. However, such fragmentation can be expected for an automatic annotation. Despite the above limitations, the transcriptome assembly shows a good N_50_ (3–3.1 Kbp) and high scaffold coverage (437–1,073x), and is thus suitable for the purpose of the present study, i.e. providing a roadmap of genomic elements differentially expressed between green and black-olive growth conditions. We will hereafter refer to this draft assembly as the “*Ca. E. dacicola* transcriptome”.

### Differential expression of *Ca. E. dacicola* genes in green and black olive-fed larvae

In order to obtain an overview of induced processes in the microbial community of the gastric caeca, we quantified bacterial gene expression by separately aligning the *Erwinia* RNA-seq reads coming from caecas of green and black olive-fed larvae against the *Ca. E. dacicola* transcriptome. The aim was to identify genes with high expression levels in either of the two time-points of fruit ripening. We will hereafter refer to over- and under-expressed genes relative to the green olive condition, with both profiles being of interest for the identification of genes relevant to the interaction of the fruit fly with its gut symbiont. The alignment of RNA-seq reads to the draft transcriptome was performed with the bacterial-specific program EDGE-pro^45^. Using the Reads Per Kilobase of transcript per Million mapped reads (RPKM) as a ‘proxy’ of expression levels, we identified 81 over-expressed and the 41 under-expressed DEGEs (differentially expressed genomic elements). Table S9 (sheets 1 and 2) lists these elements along with information on the *p-value* of differential expression we obtained from an additional analysis run with the Bioconductor package DESeq2, which has an option of treating data without biological replication^46^. DEseq2 analysis identified 7 significantly (*p-value* < 0.05) over-expressed DEGEs (Table S9, sheet 3), five of which in common with the RPKM approach.

Literature searches and functional enrichment analysis of the GO terms associated with the *Ca. E. dacicola* DEGEs revealed that different sets of processes are activated in the bacterial symbiont in green compared to black olive -fed larvae: Few processes were activated in the caeca of larvae feeding on green olives, with a strong bias for those related to genomic (ex)changes and to the metabolism of aromatic, nitrogen and nucleobase-containing compounds. These were the dominant terms (Biological Process and Molecular Function GO categories) found to be significantly enriched with an FDR *p-value* < 0.05 (Table S4). More specifically, over-expressed genomic elements were dominated (64.2%) by predicted mobile genetic elements (MGEs), transposases and integrases, virB proteins of the type IV secretion system that allows inter-bacterial and bacterial-eukaryote conjugative transfer of DNA and other macromolecules (for example, Schroder *et al*.^47^), and a topoisomerase involved in recombination or transcription. These findings indicate that, during larval growth in green olives, DNA exchanges are increased within the symbiotic community of the gastric caeca, with MGEs playing a central role in this process between and within genomes (reviewed in Aminov^48^), in agreement with previous studies^49^. The genomic exchanges taking place within the gut microbial community may be directly beneficial for the symbiont and/or the insect in the stressful environment of the green olive, *via* alterations in gene regulation, genome rearrangements and lateral gene transfer. These processes are highly dynamic in symbiotic systems, despite hundreds of millions of years of host-microbe coevolution^50^. Concurrent with the observed expression of type IV secretion system components in our study are ultrastructure studies of symbiotic bacteria in olive flies^51^. These show an extensive network of filamentous fimbriae protruding from bacterial cells and spanning into their surroundings. These fimbriae may facilitate exchange of genetic material as discussed above, or nutritive elements (see below).

Among over-expressed genes in the present study, we identified several with relevant protein functions providing support for induced biosynthesis of amino acids or fixation of nitrogen. For example, we identified three molybdopterin-guanine dinucleotide biosynthesis genes, encoding ModB, ModC and TriL/ModC proteins, and an endoribonuclease L-PSP, showing similarity to imine deaminases, a large conserved family of proteins related to amino-acid biosynthesis (for example, isoleukine biosynthesis in another enterobacterium^52^). The overexpression of the ModB-encoding gene, further supported by DESeq2 analysis (*p-value* = 0.027), might be of particular interest in this context: The ModB protein is related to the trace element molybdenum, present in nitrogenase metalo-enzymes that perform nitrogen fixation. The exact role of ModB appears controversial, even in studies of the model system *E. coli*, with roles in either biosynthesis of the molybdenum cofactor and anaerobic respiration^53^, or in molybdenum transport^54^. Notably, the ModB gene was recently identified in the genomes of two bacterial symbionts of an insect pest, and these two symbionts are phylogenetically close to *Erwinia*^55^. Another over-expressed gene for which there is statistical support from DESeq2 analysis and that is potentially related to amino-acid metabolism encodes the PvcB protein, a member of the family of Fe^2+^ α-ketoglutarate-dependent oxygenases. Recently, the PvcB protein was functionally characterized in three bacterial species, including an *Erwinia* species, and was shown to be involved in biosynthesis of secondary metabolites from aromatic amino acids^56^.

The activation of metabolic processes related to aromatic, nitrogen and nucleobase-containing compounds highlights the importance of amino-acid and nitrogen availability in the green olives, expected to be limited in this environment. Indeed, unripe olives employ a potent defence mechanism against herbivores through activation of oleuropein – their major phenolic constituent^29^. Once activated, oleuropein exhibits strong protein cross-linking and lysine-decreasing activities, eventually leading to severe reduction in protein availability and nutritional value^13^,^28^. Larvae feeding on unripe fruit are unable to complete development when devoid of *Ca. E. dacicola*. These bacteria are suspected to counteract the anti-nutritive activity of oleuropein, either by compensating for the lack of nutritionally-balanced protein source or through direct detoxification^13^,^14^,^15^. Either of these processes may require activation of amino-acid synthesis in the bacterium: *Ca. E. dacicola* has been previously demonstrated to sustain protein synthesis in adult flies by providing essential amino acids^23^. Additionally, detoxification of oleuropein may be effectively achieved into the larval gut through secretion, by the bacterium, of free amino acids that inactivate this phenolic compound, as for example glycine (Konno *et al*.^57^ and references within). Glycine inhibits the lysine-decreasing activity of (enzymatically) activated oleuropein by antagonizing the glutaraldehyde-like structure. Thus, a tempting hypothesis to be further tested is that secretion of glycine occurs *via* the type IV secretion system, previously visualized through microscopy in olive flies^51^, since a number of its components were found over-expressed in our study (Table S9). The roles of this system are highly diverse and not fully known in bacteria (reviewed in Souza *et al*.^58^), but may relate to the establishment of symbiotic interactions, as previously shown for the nitrogen-fixing bacterium *Mesorhizobium loti*^59^. Overall, a number of previous studies support that the metabolism of amino acids is an important component underlying the interaction between *B. oleae* and *Ca. E. dacicola* both in adult and larval life stages, in terms of either development and/or nutrition of *B. oleae* within the olive. Correspondingly, the activation of processes related to nitrogen metabolism as depicted by our transcriptomic survey is supported by these studies.

A different set and a higher variety of processes appeared activated in the caeca of larvae feeding on black olives based on GO enrichment analysis (Table S4): primary metabolism, most often related to the biosynthesis of basic cell-wall components (phosphoenolpyruvate carboxykinase, phosphoheptose isomerase 1, UDP-N-acetylmuramoylalanyl-D-glutamyl-2,6-diaminopimelate–D-alanyl-D-alanine ligase), amino-acid metabolism (e.g. aspartate-semialdehyde dehydrogenase, aspartate 1-decarboxylase, histidinol-phosphate aminotransferase), transport of various compounds (PtsN system for carbohydrates, amino-acid ABC-type transporter, magnesium and cobalt efflux protein CorC, permeases), heat/cold shock response (CspD, HSP33, HtrA protease/chaperone), DNA repair (exodeoxyribonuclease V beta chain, DNA ligase) and transposition (MGEs constituting 17.07% of under-expressed elements). However, statistical support for enrichment of relevant GO terms was marginal and mainly affected gluconeogenesis and biosynthesis of methionine, isoleucine, threonine and alanine, as well as a range of enzymatic activities, such as aminotransferases, isomerases, oxidoreductases, decarboxylases and carboxykinases. The overall profiles of bacterial gene expression in the black olive environment indicate that metabolism of amino acids is still highly active at this late stage of fruit ripening, but with probably different pathways being induced as compared to the green olive environment. Because obtaining the biological material for this study presents several biological and technical difficulties (gastric caeca dissection, standardized extraction of larvae from ripe and unripe olives at the same period of the year), it was not possible to robustly assess gene expression of the symbiont through use of biological replicates. Nevertheless, future RT-qPCR experiments should confirm the expression of *Ca. E. dacicola* genes identified here and contribute in identifying the exact succession of molecular events occurring at the level of amino-acid metabolism in the symbiont during olive fruit ripening.

## Concluding remarks

This study presents a joint gene expression analysis of olive fruit fly larvae and their major bacterial symbiont *Ca. E. dacicola* developing in green and black olives. In green immature olives, known to contain large quantities of the phenolic compound oleuropein, several olive fruit fly genes encoding detoxification enzymes, such as UGTs, were found significantly over-expressed. Under the same conditions in the symbiont, components of the type IV secretion system, previously associated with secretion of an inhibitor of oleuropein, were found activated. At this stage, we cannot say whether degradation of oleuropein occurs with insect or symbiont enzymes (or their cooperation), but our study provided some first evidence- for potential contributions in oleuropein degradation from both partners of the symbiotic interaction. In addition, we observed an activation of several digestive enzymes in the olive fruit fly, and of amino-acid metabolism in the bacterial partner. The above genes and processes might also relate with the ability of the olive fruit fly to feed on green olives.

Numerous olive fruit fly genes of yet unknown function were also differentially expressed. The sequencing and annotation of the *B. oleae* genome is expected to unravel the potential roles of these and additional genes in the adaptation to the olive environment. Our study further provides a first transcriptomic resource for the *B. oleae* gut microflora, recognized since long as a key player in the adaptation, especially to the green olive environment. The annotation and first comparative analysis of the bacterial draft transcriptome provide an important basis for future studies aiming to identify the functional contributions of the symbiont to the development and nutrition of the *B. oleae* larvae within the olives, especially with regards to amino-acid metabolism.

## Materials and Methods

### Microarray design

A custom Sureprint genome-wide G3 Gene Expression 8 × 60 k microarray (design ID 045129) was designed using the Agilent eArray online platform (http://earray.chem.agilent.com/earray, Agilent Technologies) based on the *B. oleae* contigs obtained by 454 pyrosequencing (BioProject accession number PRJNA195424, Sequence Read Archive: SRR799687, SRR799688), a set of 15 *B. oleae* P450 sequences obtained by degenerate PCR^10^ and 236 *B. oleae* sequences (including 13 mitochondrial genes) available at NCBI (database version of November 2012). Fungal rRNA sequences were removed from the *B. oleae* contig dataset prior to probe design^10^. For *B. oleae* contigs and sequences that showed homology (blastx^60^, e-value threshold E-3) to other arthropod proteins and for which strand orientation information was available, probe design aimed for three to four probes of 60 nt per contig with parameters set to “best probe distribution” and “no 3’ bias“. For those *B. oleae* contigs that showed homology to arthropod proteins on both strands, open reading frames (ORFs) from both strands and non-coding regions were extracted, and probes were designed aiming at 3 probes per ORF/non-coding region, and with parameters set to “best probe distribution” and “no 3′ bias”. For those *B. oleae* contigs that showed no homology to arthropod proteins on either strand, probes were designed for both the forward and reverse strand (3 probes/strand/contig, “best probe distribution”, “no 3′ bias”). In total, 59,860 probes targeting *B. oleae* transcripts were designed. Finally, we also designed 4 probes per ORF (“3’bias”, “best probe design”) for 31 *B. oleae* ORFs having a blastx hit with arthropod “housekeeping” genes (Table S1). These probes were randomly distributed in 10 copies per array and were used to measure intra-array reproducibility (“replicate non-control probe group”). The array design was submitted to the National Center for Biotechnology Information (NCBI) under the Gene Expression Omnibus (GEO)-platform format (GEO platform number GSE82037). The slide layout comprises eight arrays, allowing two comparisons of four replicates each.

### Sample collections

Unripe (green) olives of the Kalamon variety infested by *B. oleae* were collected from an olive orchard in Heraklion (Greece) in September 2012. Infested olives were transferred to the laboratory and 2^nd^ instar larvae were picked up from the mesocarp. Specimens were preserved in RNAlater (Sigma-Aldrich) and stored at −80 °C until use. Second-instar larvae from mature (black) olives were collected in January 2013 from the same olive orchard following the same procedure as described for green olives. To obtain samples from artificial diet, infested olives were collected from the same olive orchard, transferred to the laboratory and maintained in Petri dishes containing a layer of sawdust (substrate for pupation), at 25 °C, with a 16:8 h light: dark photoperiod. Upon pupation, pupae were transferred in plastic cages (8 × 8 × 8 cm) and the emerging females were allowed to lay eggs in a wax substrate. The newly hatched larvae were transferred to Petri dishes with artificial diet containing 550 ml distilled water, 20 ml extra virgin olive oil, 7.5 ml Tween-80, 0.5 g potassium sorbate, 2 g Nipagin, 20 g sugar, 75 g Brewer’s yeast, 30 g soy hydrolysate, 30 ml hydrochloric acid 2 N and 75 g cellulose powder^61^. Larvae were collected after they had reached the 2^nd^-instar stage and were stored at −80 °C upon treatment with RNAlater (Ambion).

Gastric caeca samples were prepared as follows: Caeca were extracted from 3^rd^-instar larvae that developed in olives in the laboratory. To rear larvae, wild flies were obtained as pupae from field-collected olives during December 2013. Following ecdysis and for the next 14 days females were maintained on a liquid diet of sucrose and yeast hydrolysate as previously described^13^. On the 8th day post-ecdysis, males were introduced into the cage to allow females to mate, and subsequently females oviposited in unripe (green) and ripe (black) olives (Cv. “Barnea”). Olives were picked 5 days earlier and maintained at 4 °C.

Ripe (n = 15) and unripe (n = 25) olives containing eggs were incubated at 25 °C for 10 days, after which the 7–8 day old, 3^rd^-instar larvae were extracted from the fruit. After external rinsing, larvae were dissected and their gastric caeca (n = 10 for each fruit condition: unripe and ripe) containing the bacteria were preserved in 500 μl RNAlater (Ambion) overnight at 4 °C. The excess solution was decanted the following day and samples were frozen at −80 °C.

### RNA preparation for microarray experiments

Samples were collected from 2^nd^-instar larvae fed upon green and black olives and artificial diet in four biological replicates. For each replicate, total RNA was extracted from 20 pooled individuals, using the RNeasy mini kit (Qiagen). RNA samples were treated with Turbo DNA-free (Ambion) to remove any contaminating genomic DNA. RNA quantity and quality were measured on a Nanodrop ND-1000 spectrophotometer (NanoDrop Technologies) and by running a 1.5% agarose gel. One hundred nanogram of RNA was used to generate Cy3- and Cy5-labeled cRNA following the Low Input Quick Amp Labeling kit (Agilent Technologies). RNA spike-in controls (Agilent Technologies) were added prior to the reverse transcriptase reaction step in the protocol. The labeled cRNA was purified with the RNeasy mini kit (Qiagen). RNA and cyanine concentration was measured on a Nanodrop ND-1000 spectrophotometer (NanoDrop Technologies).

### Gene expression microarray set-up and analysis

Two major hybridization schemes were followed: Four Cy5-labelled replicates of larvae fed on green olives were hybridized with either four Cy3-labelled replicates of larvae fed on black olives or either four Cy3-labelled replicates of larvae reared on artificial diet. Cyanine-labeled cRNA was pooled and hybridized to the custom-made Sureprint G3 8 × 60 K arrays using the Gene Expression Hybridization kit (Agilent Technologies). Slides were placed in a rotating (20 rpm) hybridization oven at 65 °C for 17 hours. Following hybridization, slides were washed using the Gene Expression Wash Buffer kit (Agilent Technologies) and scanned by an Agilent Microarray High-Resolution Scanner using default settings for 8 × 60 K GE microarrays. A raw image file for each array (accessible at GSE82037) was extracted using the Agilent Feature Extraction Software (GE2_107_SEP09 protocol). The raw Cy5and Cy3 (RG) intensity values were transferred to the limma framework for statistical processing^62^. Quality of the data throughout the limma pipeline was verified using arrayQualityMetrics^63^. Background-corrected RG data (normexp, 50) were used to generate the MA (M; log ratio and A; mean average) data^64^. MA data were subsequently optimized by normalization using loess and Aquantile^62^. A linear model was fitted to the processed data to generate three sets of relative gene expression levels: larvae fed on green olives *vs.* larvae fed on black olives, larvae fed on green olives *vs*. larvae fed on artificial diet and larvae fed on black olives *vs.* larvae fed on artificial diet. Significant differential expression was identified by applying empirical Bayesian statistics, using 0.05 and 1 as cut-offs for the FDR-corrected *p*-value and log_2_FC, respectively. PCA was performed on the transcript levels of 17,797 contigs (FDR-corrected *p-value* < 0.05 in at least one condition) using the prcomp command from the *stats* package. Two types of gene set analyses were performed to identify significant enrichment of Gene Ontology (GO) terms in the lists of significantly over- and under-expressed genes. For these analyses, we used the GO annotations of the olive fruit fly transcriptome^10^. Fisher’s Exact tests (“two-tailed”), combined with Benjamini-Hochberg False discovery rate (FDR) correction for multiple testing^40^ were performed for the identification of over- and under-represented GO terms via the Blast2GO software^65^. Significant GO terms (*p*-value < 0.05) were ranked by *p*-values. Biological process GO enrichment was additionally examined by using the differential expression-associated statistics obtained through linear modeling in a distinct directional gene set analysis (PAGE)^66^ using the *piano* package. *B. oleae* expression data and array platform have been uploaded to the Gene Expression Omnibus with accession number GSE82037.

### Microarray validation by RT-qPCR

Selected detoxification and digestive genes were validated using quantitative reverse transcription PCR (RT-qPCR). Primers were designed using the Primer – BLAST online analysis software (http:/www.ncbi.nlm.nih.gov/tools/primer-blast/) based on cDNA sequences retrieved from previous work^10^. These primers are listed in Table S2. Reverse transcription was performed on 2 μg of RNA (used for cRNA production) for each condition, using Superscript III (Invitrogen Life Technologies) following the manufacturer’s instructions. PCR reactions of 25 μl were performed on a MiniOpticon two-color Real-Time PCR detection system (BioRad), using 0.20 μM of primers and KapaSYBR FAST qPCR master mix (Kapa- Biosystems). A 5-fold dilution series of pooled cDNA was used to assess the efficiency of the qPCR reaction for each gene-specific primer pair. A no-template control (NTC, #2587) was also included to detect contamination. Melting-curve analysis was performed to test the specificity of amplicons. Experiments were performed in 4 biological replicates for each contig/gene. The expression level of each target gene was normalized to that of the contigs encoding for the 40S ribosomal protein (GAKB01005984.1) and beta-actin (GAKB01001968.1), and relative expression levels were calculated according to Pfaffl^67^.

### RNA-sequencing of gastric caeca samples and taxonomical mapping

RNA isolation, library preparation and Illumina sequencing were performed by LGC Genomics GmbH (Berlin, Germany). Briefly, total RNA was extracted from one sample of 15 3^rd^-instar symbiotic larvae developing in unripe olives (“green” sample) and a second sample of 13 3^rd^-instar symbiotic larvae developing in ripe olives (“black” sample). rRNA-depleted, random-primed stranded libraries were constructed with the NuGEN protocol. Random priming allowed minimizing the sequences coming from the insect, since eukaryotic RNAs are characterized by polyA-tails. The libraries were sequenced in paired-end mode (2 × 300 bp) on the Illumina Miseq platform (V3). Demultiplexing of reads and adapter clipping was performed using Illumina’s CASAVA data analysis software, and clipped reads with a length of less than 20 bases were discarded. A total of 15,093,728 and 11,852,040 adapter-clipped reads were finally obtained for the “green” and “black” sample, respectively. Raw sequence reads from RNA sequencing of gastric caecas from green and black olive-fed larvae are available through GenBank, linked to the project PRJNA325102, under the accession numbers SRR3656965 and SRR3656965, respectively. Quality control analysis was performed using FastQC software^68^. Overlapping paired-end reads (R1, R2) were merged to obtain longer single-end fragments using the open-source utility BBMerge version 5.1 (available within the BBTools package http://jgi.doe.gov/data-and-tools/bbtools/, accessed 02/2015) with default parameters. Prior to assembly of the transcriptome, merged (“single”) and unmerged (“paired”) reads were taxonomically classified. For this purpose, we used the program Kraken v0.10.5-beta^69^ to perform an exact alignment of k-mers present in the reads against the RefSeq collection of completed microbial genomes available at the NCBI database and available within Kraken (data downloaded 25/11/2014). Since, at the time, this collection contained genomic DNA and plasmid sequences from only 7 *Erwinia* strains (*Erwinia sp.* Ejp617, *E. amylovora* ATCC 49946, *E. amylovora* CFBP1430, *E. billingiae* Eb661, *E. pyrifoliae* DSM 12163, E*. pyrifoliae* Ep1 96, and *E. tasmaniensis* Et1 99), we used the BWA-MEM algorithm^70^ to perform alignments of the same reads against a set of *Erwinia* sequences compiled from NCBI data in May 2015 and containing all publicly available ribosomal sequences of *Ca. E. dacicola* and genomic sequences of an additional 11 *Erwinia* strains: *E. toletana* DAPP-PG 735 (acc: GCA_000336255.1). *E. carotovora* subsp. *atroseptica* (acc: BX950851.1), *E. pyrifoliae* strain WT3 (hrp pathogenicity island, acc: DQ180962.2), *E. sp.* Ejp 556 (pEJ30 plasmid, acc: NC_004834.1| & AY255829.1), *E. amylovora* strain MR1 (pEA29 plasmid, acc: NG_035358.1 & AF264949.1), *E. amylovora* strain NBRC 12687 (acc: BAYW01000003.1), *E. typographi* strain M043b (acc: GCA_000773975.1), *E. mallotivora* strain BT-MARDI (acc: GCA_000590885.1), *E. tracheiphila* strains PSU-1 and BuffGH (acc: GCA_000404125.1 & GCA_000975275.1) and *E. oleae* strain DAPP-PG531 (acc: GCA_000770305.1). This allowed obtaining a more exhaustive dataset of reads potentially coming from the genome of *Ca. E. dacicola*. The BWA alignments were performed using default parameters, and only reads that aligned with a quality score of 30 or above were retained.

A phylogenetic tree of 16 S rRNA sequences of *Ca. E. dacicola* and related gamma Proteobacteria inhabiting the gut of *B. oleae* was constructed. We selected sequences of a length higher than 700 bp with no unknown-length gaps (Ns). The sequences were aligned with MUSCLE^71^, and Bayesian trees were created from the alignment with the program BEAST^72^. The trees from BEAST were summarized using the maximum product of the posterior clade probabilities criterion with a threshold of 0.7.

### Assembly, functional annotation and enrichment analysis of the *Candidatus Erwinia dacicola* transcriptome

Assembly of the *Ca. E. dacicola* transcriptome was performed *de novo* on the reads that were classified as *Erwinia* from the “green” and “black” samples, using the program rnaSPAdes v.0.1.0^36^. rnaSPAdes was run in single-cell mode with tested k-mer sizes 21,33,55,77,99,127, following the developers’ recommendations for Illumina long-read RNA-seq data. This Transcriptome Shotgun Assembly (TSA) project has been deposited at GenBank under the accession GEZS00000000. The version described in this paper is the first version. Note that for the purpose of the TSA submission, contigs of length <200 bp were removed; none of the genomic elements discussed below as differentially expressed was predicted from the removed contigs. The full transcriptome assembly containing all contigs, along with its annotation, are available in Table S8. For the annotation of the assembled transcriptome, we used the RAST platform^38^ with bacterial-specific parameters. Functional enrichment analysis in the two lists of over- and under- expressed genes was performed with the Blast2GO tool^65^. Briefly, blastx searches against the nr collection of NCBI were performed for all nucleotide sequences, and GO term annotations^73^ were retrieved for high-scoring (>1.0E-3) segment pairs that covered at least 70% of the longitude of the corresponding blastx hits. Only GO terms known to be part of the taxonomy of Bacteria were selected for further analysis. Identification of significantly over- and under-represented GO terms was performed as with the olive fly data.

### Differential gene expression analysis of the symbiont

Alignment of RNA-seq reads to the draft transcriptome was performed with EDGE-pro v.1.3.1^45^. This program is specifically designed for bacterial gene expression analysis, and takes into account the absence of introns and the overlapping nature of bacterial genes, while it provides different options for the treatment of multi-mapping reads. Counts and RPKM values for differential gene expression analysis were obtained by running EDGE-pro separately on “paired” and ‘single’ reads from each condition (green, black), with the following parameters: −w 250; −c 6; −m 35 and −M 588 (for mapping of ‘paired’ reads only); −l 252, 262, 225 and 239 for ‘paired’ reads from the “black” and “green” sample, and ‘single’ reads from the “black” and “green” sample, respectively. The value of −w was set to 250 based on the average length of our Illumina reads, while the −c (minimum average gene coverage for a gene to be considered expressed) was set to the double of the default value, based on the fact that RPKM values estimated by EDGE-pro for paired-end data are artificially doubled; this issue is discussed by the developers of the program^37^. Values of −l, −m and −M were based on the average length of reads and the average insert size of our libraries, as estimated by BBMerge (see above). EDGE-pro was run twice, once with the option of giving partial credit (counts) to all genes where a read may map (−n 0), and the second time with the option of arbitrarily assigning all multi-mapping reads to one gene (−n 1); these options were tested to assess the robustness of differential expression for repetitive sequences in the genome. In addition, the Bowtie2 command internally run by EDGE-pro was edited to account for the orientation of reads, by adding the parameter −fr. As ‘genome’ for each run, we designated the concatenated (with 100 Ns separating different contigs) *de novo* transcriptome assembly of *Ca. E. dacicola*, while −p and −r annotation files came from manual editing of RAST annotation files, in agreement with the “ptt” format required by EDGE-pro. The EDGE-pro output was converted to the format used by the Bioconductor package DESeq2^45^ using the edgeToDeseq.perl script within EDGE-pro, while average coverage and RPKM values for each condition were manually calculated in Excel by summing the results on ‘paired’ and ‘single’ reads data. Genomic elements that showed zero RPKM values in either condition were excluded from further analysis. We defined as differentially expressed the elements that showed RPKM values ≥4 since biological replicates were not available for a full statistical analysis.

DESeq2 analysis was performed on the estimated ‘counts’ for each gene in each condition in R^74^. Following negative binomial fitting of the data, two statistical methods were used for assessing significance of differential expression, likelihood ratio (LRT) and Wald tests. Because it is not possible to estimate the dispersion of counts around the expected value for each group in unreplicated experiments, we followed the strategy outlined in Anders and Huber^46^, wherein all the samples are considered as replicates of a single group for the estimation of dispersion. This approach is conservative, since some overestimation of the variance may be expected. We considered as differentially expressed these elements that showed in DESeq2 analysis a *p*-value < 0.05 in two different statistical tests, Wald and LRT, and in both EDGE-pro runs with regards to treatment of multi-mapping reads. It should be noted that significance of the statistical tests was null after multiple testing correction.

## Additional Information

**How to cite this article**: Pavlidi, N. *et al*. Transcriptomic responses of the olive fruit fly *Bactrocera oleae* and its symbiont *Candidatus Erwinia dacicola* to olive feeding. *Sci. Rep.* **7**, 42633; doi: 10.1038/srep42633 (2017).

**Publisher's note:** Springer Nature remains neutral with regard to jurisdictional claims in published maps and institutional affiliations.

## Supplementary Material

Supplementary Information

Supplementary Table S1

Supplementary Table S2

Supplementary Table S3

Supplementary Table S4

Supplementary Table S5

Supplementary Table S6

Supplementary Table S7

Supplementary Table S8

Supplementary Table S9

## Figures and Tables

**Table 1:** Draft transcriptome assembly of *Candidatus Erwinia dacicola.*

Feature (all – scaffolds > 200 bp)	Value
# scaffolds	2,161–1,731
Average scaffold length (bp)	1,281–1,559
N_50_ (bp)	3,008–3,175
Total length (bp)	2,769,894–2,699,562
Average scaffold coverage (x)	1,073–437
# coding genes	3,206
# tRNAs	44
Average coding gene length (bp)	540

N_50_ is a weighted median statistic such that 50% of the entire assembly is contained in scaffolds equal to or larger than this value. #: number.
