# Supplementary material for: Transcriptomic responses of the olive fruit fly Bactrocera oleae and its symbiont Candidatus Erwinia dacicola to olive feeding
Source: Sci Rep. 2017 Feb 22;7:42633. doi: 10.1038/srep42633 (PMC5320501; doi:10.1038/srep42633)
Supplement: Supplementary Information [file srep42633-s1.pdf]

Supplementary Information

Transcriptomic responses of the olive fruit fly *Bactrocera oleae* and its symbiont *Candidatus Erwinia dacicola* to olive feeding

Nena Pavlidi[1,2#], Anastasia Gioti[1#], Nicky Wybouw[2], Wannes Dermauw[3], Michael Ben-Yosef[4], Boaz Yuval[4], Edouard Jurkevich[5], Anastasia Kampouraki[1,6], Thomas Van Leeuwen[2,3], John Vontas[6, 7*]

[1] Department of Biology, University of Crete, 71409 Heraklion, Greece

[2] Institute for Biodiversity and Ecosystem Dynamics (IBED), University of Amsterdam (UvA), Science Park 904, 1098 XH Amsterdam, The Netherlands

[3] Department of Crop Protection, Faculty of Bioscience Engineering, Ghent University, B-9000, Ghent, Belgium

[4] Department of Entomology, The Hebrew University of Jerusalem, Rehovot 76100, Israel

[5] Department of Plant Pathology and Microbiology, The Hebrew University of Jerusalem, Rehovot 76100, Israel

[6] Institute of Molecular Biology & Biotechnology, Foundation for Research & Technology Hellas, 100 N. Plastira Street, GR-700 13, Heraklion Crete, Greece

[7] Laboratory of Pesticide Science, Department of Crop Science, Agricultural University of Athens, 11855-Athens, Greece.

# equal contribution

* corresponding author

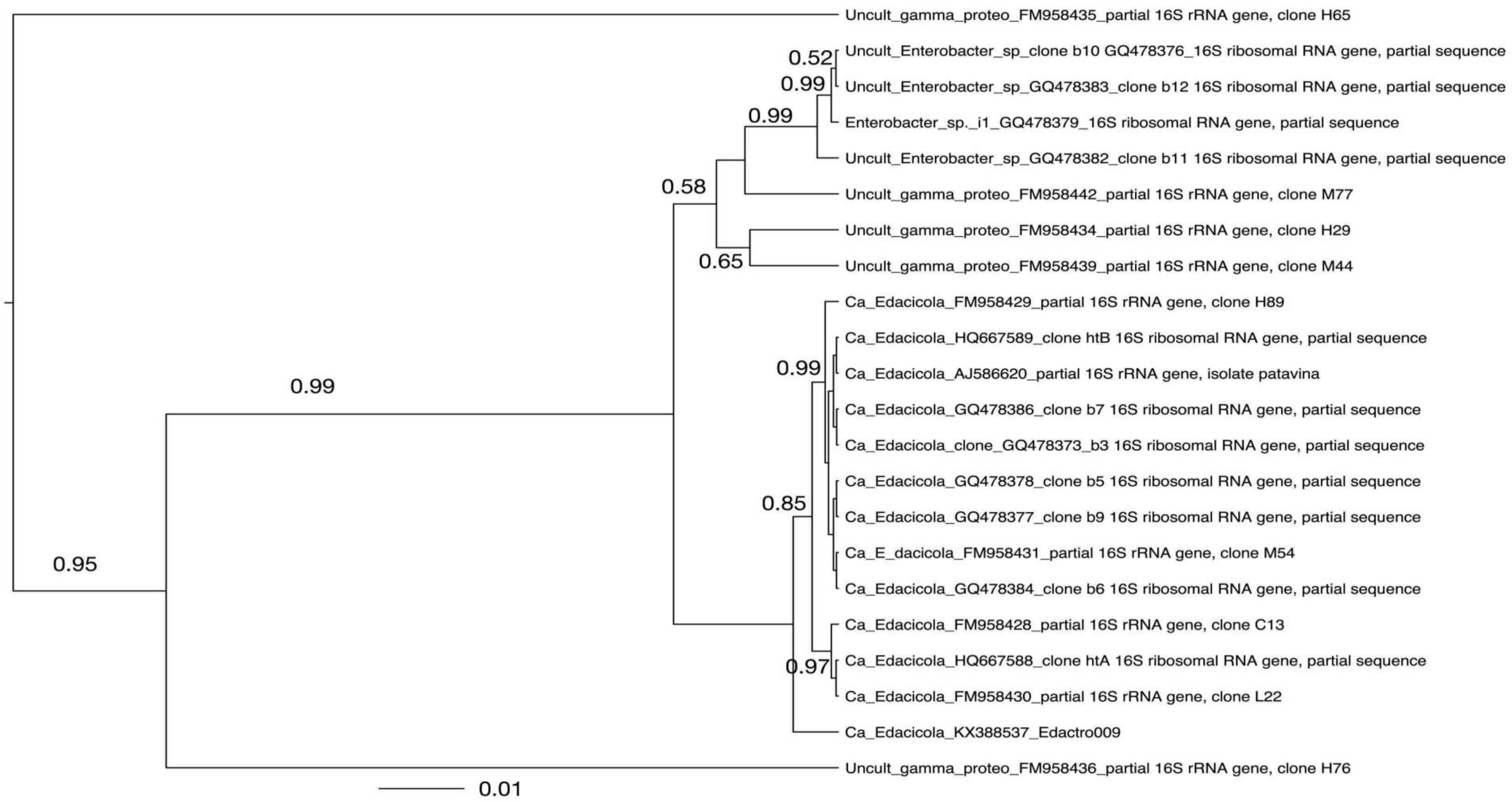

Figure S1. Phylogenetic tree of 16S rRNA sequences of *Ca. E. dacicola* (KX388537) and related gamma Proteobacteria inhabiting the gastric caeca of *B. oleae.* The sequences were retrieved from NCBI (accession numbers noted in the name of each sequence). Bayes posterior probabilities > 0.5 are noted in the tree.

Table S1. *B. oleae* ORFs having a blastx hit with arthropod "housekeeping" genes.

Table S2. qPCR primers used in this study.

Table S3. *B. oleae* contigs differentially expressed between larvae fed upon green olives and artificial diet.

Table S4. GO terms enriched in *B. oleae* and *Ca. E. dacicola* differentially expressed genes based on at least one of the three criteria: p-value from Fisher tests, adjusted p-value from Fisher tests, and adjusted *p*-value from PAGE tests.

Table S5. *B. oleae* contigs differentially transcribed between larvae fed upon black olives and artificial diet.

Table S6. *B. oleae* contigs differentially transcribed between larvae fed upon green olives and black olives.

Table S7. Results of taxonomical mapping (with the program Kraken) of the RNA-sequencing reads coming from caecas of larvae fed on a) green and b) black olives. Unclassified reads are in majority of eukaryotic origin, since the Kraken database is microbial.

Table S8. *Ca. E. dacicola* draft transcriptome assembly sequence and annotations based on RAST. Note that that locations refer to an assembly containing 100Ns between contigs, for the purpose of the EDGE-pro analysis.

Table S9. *Ca. E. dacicola* predicted genomic elements differentially expressed between larvae fed upon green olives and black olives (a) over-expressed based on the RPKM approach, (b) under-expressed based on the RPKM approach and (c) over-expressed based on the DESeq2 approach. All three lists are sorted by descending expression ratio (green/black for over-, black/green for under-).
